# Effectiveness of a trunk-wearable neuromuscular electrical stimulation device in postpartum women with diastasis rectus abdominis: A prospective randomized controlled trial

**DOI:** 10.1017/wtc.2025.10035

**Published:** 2025-12-11

**Authors:** Linan Zheng, Yunfeng Zhang, Congyu Jiang, Kai He, Yulan Zhu

**Affiliations:** 1 Department of Rehabilitation, Huashan Hospital Fudan University, China; 2 General Surgery, Huashan Hospital Fudan University, China

**Keywords:** diastasis rectus abdominis, exercise therapy, low-back pain, neuromuscular electrical stimulation, postpartum trunk weakness, wearable device

## Abstract

Diastasis of rectus abdominis (DRA) is a common pathological condition in postpartum rehabilitation, but with limited treatment strategies. This study aimed to explore the effect of using a trunk-wearable neuromuscular electrical stimulation (NMES) device on postpartum women with moderate and severe DRA. A total of 84 postpartum women with an inter-rectus distance (IRD) of ≥3 cm were randomly assigned to two equal groups. The treatment group received a trunk-wearable NMES device and exercise therapy, whereas the control group received exercise only. We measured IRD and calculated treatment response proportion, improvement of trunk muscle strength, and low-back pain in both groups. Additionally, we evaluated quality of life (QoL) using the SF-36 questionnaire and Hernia-related Quality of Life Survey (HerQLes). Statistical analysis was performed using SAS 9.4. After 8-week treatment, the IRD of the umbilical (M3) sector showed a greater reduction in the treatment group (−10.6 [−17.9 to −3.3]%, *p* < 0.05). Patients in the treatment group had higher treatment response proportions (*p* = 0.0031 and *p* = 0.0010, W2 and W3, respectively). Additionally, the treatment group had higher Janda assessment scores and greater reduction in low-back pain (both *p* < 0.0001). QoL evaluation indicated greater improvements in the SF-36 questionnaire (pain and role-emotional scales,*p* < 0.05) and HerQLes (*p* < 0.0001) in the treatment group. The application of a trunk-wearable NMES device on DRA patients, accompanied by exercise therapy, significantly reduced IRD and increased the treatment response proportion. Moreover, we observed positive improvements in trunk muscle strength, low-back pain, and QoL.

## 1. Introduction

Diastasis of rectus abdominis (DRA) is a common pathological condition in postpartum rehabilitation and reconstructive surgery. The incidence of DRA at 6 months and 1 year following delivery in a primiparous woman is reported to be 45.4% and 32.6% (Bø et al., 2015; Fernandes da Mota et al., 2015; Sperstad et al., 2016). An observed 2–3-cm separation between the rectus abdominal muscles is considered mild diastasis, 3–5-cm as moderate diastasis, and >5 cm as severe diastasis, classified as W1, W2, and W3, respectively, based on the proposal by the German Hernia Society (DHG) and International Endohernia Society (IEHS) (Reinpold et al., 2019). For diagnosis in clinical practice, ultrasonography (US) is recommended to measure inter-rectus distance (IRD) in postpartum women with DRA (Pirri et al., 2019).

DRA causes an imbalance between the strength and length of the abdominal wall muscles and the associated altered fascial tension, which may result in several trunk symptoms, including a decrease in core strength, abnormal muscle morphology, lumbo–pelvic pain, and deterioration of health-related quality of life (QoL), particularly physical health (Taylor et al., 2018; Benjamin et al., 2019). One elastography study used shear wave velocity (SWV) to reflect the structure and organizational resilience of abdominal wall muscles, implying that patients with DRA may also have multiple trunk muscle atrophy, dysfunction, and comprehensive coordination disorder (He et al., 2021).

Neuromuscular electrical stimulation (NMES) is a type of physical therapy that applies electric current to muscles and nerves to produce muscle contractions and simulate the exercise process, which is frequently used to strengthen postoperative muscle function recovery and prevent waste muscle atrophy in rehabilitation (Nussbaum et al., 2017; Insausti-Delgado et al., 2020; Zhao et al., 2022). An advanced study showed that NMES is effective for treating patients with mild DRA (mean IRD < 3 cm) on the basis of exercise therapy (Kamel and Yousif, 2017). Therefore, this study aimed to investigate the effect of a trunk-wearable NMES device applied to patients with DRA, particularly those with moderate and severe diastasis.

## 2. Materials and methods

**Figure 1.**
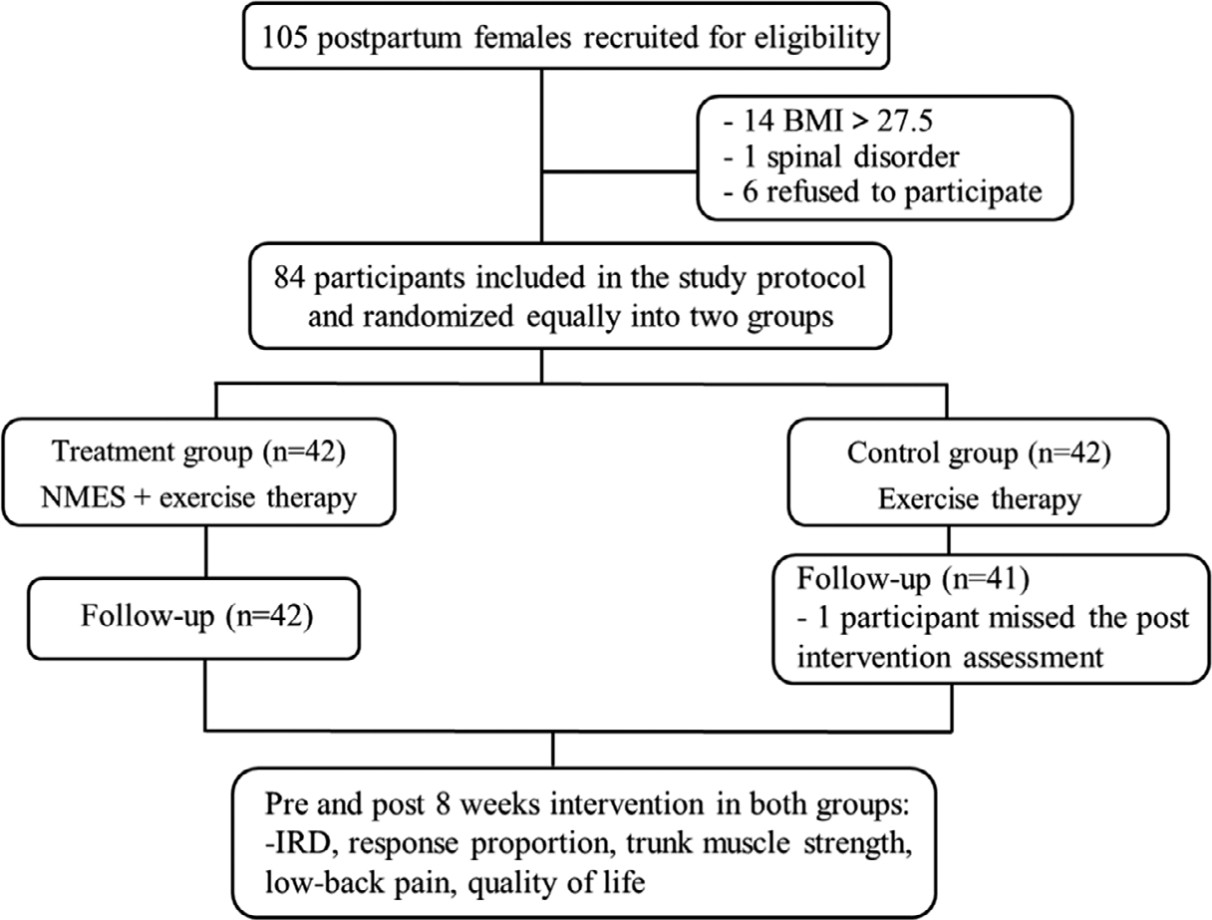
Flowchart of the study. Abbreviations: NMES, neuromuscular electrical stimulation; BMI, body mass index; IRD, inter-rectus distance.

This was a prospective randomized controlled single-blind study approved by the Huashan Hospital Ethics Committee (No. KY2020–1199) and registered in China Clinical Registry Center (No. ChiCTR2100042858, 30/01/2021). From June 2020 to June 2021, all participants were selected according to the following criteria and randomly divided into the control and treatment groups at a 1:1 ratio. Randomization was based on a computer-generated block allocation schedule. Participants were recruited for eligibility from the outpatient clinic at Huashan Hospital (Figure 1). The following were the enrollment criteria: postpartum women with DRA and an M3 width of ≥3 cm, aged between 18 and 45 years, and > 3 months and < 12 months following delivery. The following were the exclusion criteria: ongoing pregnancy, pregnancy-related complications, spinal disorders or lower limb deformities that may hinder the performance of abdominal exercises, American Society of Anesthesiologists score ≥ 3, body mass index (BMI) > 27.5 kg/m (Sperstad et al., 2016), and a US revealing abdominal recti muscle fibrosis.

For 8 weeks, both groups performed exercise therapy three times per week, and the treatment group additionally received a trunk-wearable NMES application for 30 min daily. We followed up with the patients after 8 weeks and tested the IRD using US; subsequently, we assessed the change in trunk muscle strength, low-back pain, and the QoL, and compared the data for further statistical analysis. Each patient signed an informed consent to declare their agreement to take part in this study.

### 2.1. Evaluative procedures

Evaluations were performed before and after the 8-week treatment in both groups. The assessor was blinded regarding the group’s assignment and was not involved in the treatment application. Patients with DRA underwent B-mode US examination of the IRD using a high-end scanner (Aixplorer, Supersonic Imagine, France) (He et al., 2021). The primary efficacy assessment was the proportion of patients having at least one level reduction in the IRD in the M3 sector based on the proposal by the DHG and IEHS (Reinpold et al., 2019). The trunk muscle strength, low-back pain, and QoL were evaluated using the Janda assessment, pain numerical rating scale (NRS), and short-form 36 (SF-36) questionnaire and Hernia-related Quality of Life Survey (HerQLes) (Cordier et al., 2018; Benjamin et al., 2019).

### 2.2. Treatment procedures

#### 2.2.1. Exercise therapy

**Figure 2.**
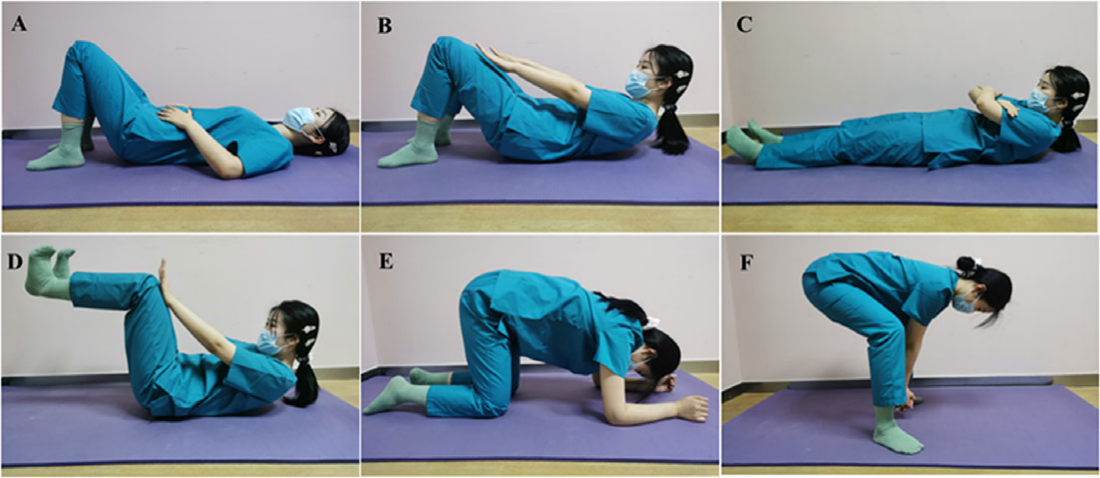
The exercise therapy was performed by both groups. (a) TrA activates breathing exercises, (b) upper RA contraction movement, (c) middle RA contraction movement, (d) RA resistance movement, (e) kneeling back-lift movement, and (f) stretching training.

Both groups performed exercise therapy three times per week for 8 weeks at the outpatient clinic department. The exercise therapy involved the use of transversus abdominis (TrA)-activating breathing exercises, core stability exercises, and stretching training (Figure 2). Participants were asked to perform the set of exercise programs for 20 repetitions, with each repetition consisting of a 5-s contraction or stretching followed by 10-s relaxation. Moreover, they were advised to repeat the same exercise program daily as a home routine program (Thabet and Alshehri, 2019).

#### 2.2.2. NMES

A trunk-wearable NMES device (ECH Medical Instrument Co., Ltd, Shanghai, China) was used only for the treatment group. It was initially applied for 30 min, followed by exercise therapy to take advantage of the improved muscle recruitment. Before starting the treatment, participants were asked to evacuate the bladder for comfort and relaxation. The electrode patch areas were cleaned with alcohol to remove debris on the skin and decrease resistance to the electrical current.

**Figure 3.**
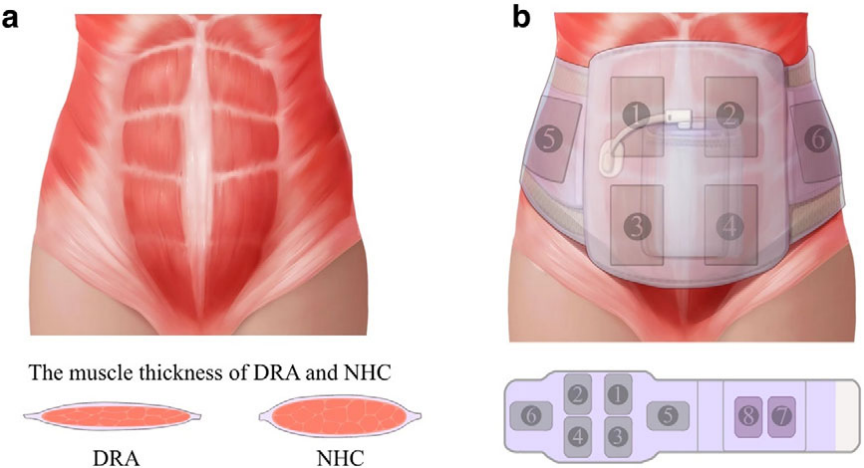
The trunk-wearable NMES device for the DRA participants. (a) The contrast of the muscle thickness of DRA and normal healthy control (NHC) and (b) the appearance of the abdominal belt and the treatment sites on the participants.

The six rectangular electrodes (numbers 1–6) used for stimulation were applied bilaterally for the external oblique (EO) muscle, upper and lower RA, and heating pads were intended to used bilaterally for quadratus lumborum (QL) at the site of numbers 7 and 8 to relieve low-back pain if necessary (Figure 3), while the site of numbers 7 and 8 were not used in this study. The rectangular electrode was made of hydrogel (Dongli Medical Technology Co., Ltd, Shaoxing, China). The parameters used in this study were a frequency of 5–35 Hz, a pulse width of 600 μS, biphasic alternating current, constant pressure control, output current of 30-50 mA and an on–off ratio of 2:1 for a total stimulation time of 30 min. The intensity was gradually increased until a good muscle contraction could be observed, and participants did not feel uncomfortable. They were subsequently instructed to relax their abdominal muscles during the NMES treatment. The diagram of a patient wearing the trunk-wearable NMES device was shown in Supplementary Materials (Supplementary Figure S1).

### 2.3. Statistical methods

Thirty-five patients per group were targeted for enrollment. Assuming that 15% and 50% of the patients in the control and treatment groups, respectively, achieve the primary efficacy of IRD improvement, this sample size provides 85%. The significant merge value for the difference between groups is *α* < 0.05.

All statistical analyses were performed using SAS 9.4 (SAS Institute, Inc., Cary, NC, USA). Binomial variables, that is, the proportion of patients achieving a one-level reduction from the baseline, will be analyzed using Fisher’s exact method by stratification according to the baseline classification of the width of rectus diastasis. Normal approximation will be applied to the estimation of a two-sided 95% confidence interval (CI) of the difference between the two groups. Continuous efficacy measurements, including pain NRS, Janda assessment, the percentage of the width of rectus diastasis, and the percentage of the SF-36 questionnaire and HerQLes assessment, will be analyzed using a covariance model with least square mean (LSM) adjustment. Since multiple dimensions of the SF-36 involved multiple hypothesis tests, we applied Bonferroni correction for the type I error, using 0.05/8 = 0.0063 as the level of significance. Factors include the study groups and baseline observed value. In the width of rectus diastasis and SF-36, 1 point will replace the 0 observed value to calculate the percentage of the observation at the 8th week of the treatment relative to the baseline.

## 3. Results

In this study, a total of 84 postpartum women were enrolled and randomly assigned to the treatment and control groups; however, one patient in the control group missed the intervention assessment. The remaining 83 participants’ demographic and baseline characteristics are summarized in Table 1.

**Table 1.** Demographic and baseline characteristics

	Control	Treatment	*p* value
N	41	42	
Ages (year), mean ± SD	34.0 ± 3.44	32.6 ± 3.09	0.0459^a^
Height (cm), mean ± SD	162.3 ± 5.08	162.0 ± 4.60	0.7499^a^
Weight (kg), mean ± SD	57.1 ± 8.42	56.9 ± 7.59	0.8997^a^
BMI(kg/m^2^), mean ± SD	21.7 ± 2.89	21.7 ± 2.63	0.9835^a^
Productions, *n* (%)			0.7414
1	22(47.8)	24(52.2)	
2	19(52.8)	17(47.2)	
3	0(0.0)	1(100.0)	
Gestations, *n* (%)			0.9489
1	18(52.9)	16(47.1)	
2	17(47.2)	19(52.8)	
3	4(50.0)	4(50.0)	
4	2(40.0)	3(60.0)	
The last delivery mode, *n* (%)			0.4949^b^
Caesarean	25(46.3)	29(53.7)	
Vaginal	16(55.2)	13(44.8)	
Multiple or single birth, (%)			0.6758^b^
Single	39(50.6)	38(49.4)	
Multiple	2(33.3)	4(66.7)	
Pregnancy complications, *n* (%)			0.0911^b^
No	30(44.8)	37(55.2)	
Gestational hypertension	2(100.0)	0(0.0)	
Gestational diabetes	9(69.2)	4(30.8)	
Others	0(0.0)	1(100.0)	
Fetal weight (kg), mean ± SD	3.4 ± 0.53	3.2 ± 0.48	0.0393^a^
Maternal weight (kg), mean ± SD			
Pre-pregnancy weight (kg), mean ± SD	53.9 ± 6.54	54.5 ± 7.81	0.7286^a^
Parturient weight (kg), mean ± SD	67.0 ± 8.15	67.1 ± 8.52	0.9411^a^
Changes (%), mean ± SD	24.7 ± 9.65	23.7 ± 8.19	0.6382^a^
Months between delivery to the first postpartum visit, mean ± SD	6.3 ± 2.52	5.7 ± 2.70	0.2607^a^

Abbreviation: SD, standard deviation.

a Student’s *t*-test.

b Fisher’s exact test.

**Figure 4.**
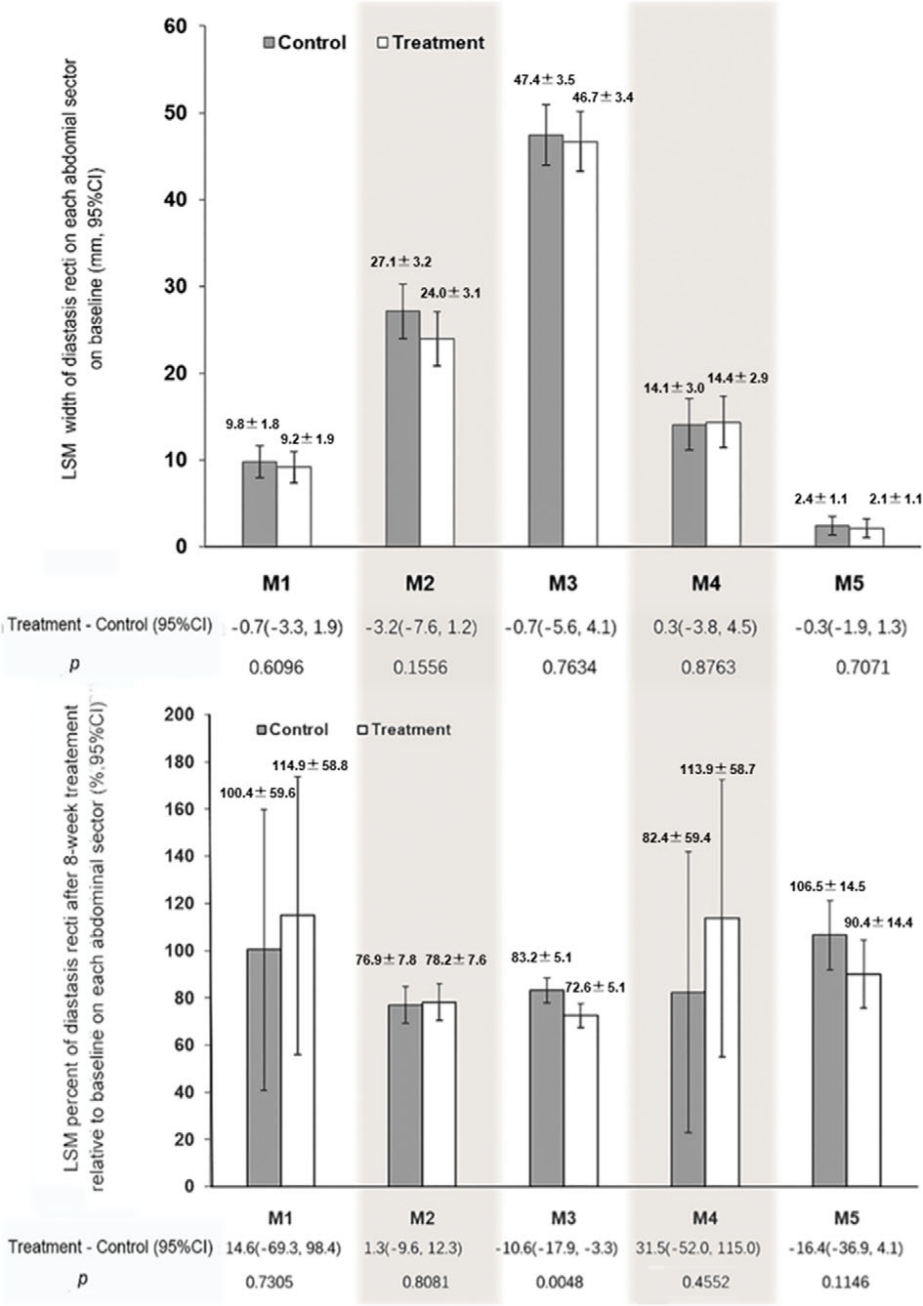
LSM IRD at baseline and the percentage relative to the baseline at the 8^th^ week of treatment on each abdominal sector. Above: the IRD (mm) at baseline; bottom: the percentage of the width relative to the baseline at the 8th week of treatment. Abbreviations: LSM, least square mean; CI, confidence interval. M1, subxiphoidal sector; M2, epigastric sector; M3, umbilical sector; M4, infraumbilical sector; M5, suprapubic sector.

At the 8th week of treatment, compared with those receiving exercise therapy only, the percentage of the IRD on the M3 sector relative to the baseline decreased in participants receiving exercise therapy combined with the trunk-wearable NMES application (treatment group, 72.6 [95% CI: 67.4–77.7]% versus control group, 83.2 [78.0–88.3]%), and the difference was −10.6 (−17.9 to −3.3)% (*p* < 0.05). No significant differences were observed in other abdominal sectors (Figure 4). The changes for IRD adjusted for baseline differences between groups (age, fetal weight) are shown in the Supplementary Materials (Supplementary Table S1).

At the 8th week of treatment, among patients with moderate DRA, the response proportions were 16 of 28 (57.1%) and 6 of 32 (18.8%) in the treatment and control groups, respectively. The response proportion difference (95% CI) was 38.4 (15.6–61.2)% (*p* = 0.0031). For patients with severe DRA, the proportions were 13 of 14 (92.9%) and 2 of 9 (22.2%) in the treatment and control groups, respectively, and the difference was 70.6 (40.3–100.0)% (*p* = 0.0010, Table 2). At baseline, the LSM pain NRS between the two groups was not significantly different, whereas at the 8th week of treatment, the LSM pain NRS was significantly lower in the treatment group than in the control group (*p* < 0.0001 and *p* = 0.0104) for both lumbago and anterior abdominal pain. Moreover, regarding the Janda assessment, the treatment group had a significantly higher abdominal muscle strength score than the control group (*p* < 0.0001) at the 8th week of treatment, but not at baseline (Table 3).

**Table 2.** Proportion of responders at the 8th week of treatment

Classification of rectus diastasis	Control		Treatment		Comparison (95%CI)^a^	*p* ^b^
	*N*	Responders (%)	*N*	Responders (%)		
Moderate	32	6(18.8)	28	16(57.1)	38.4(15.6, 61.2)	0.0031
Severe	9	2(22.2)	14	13(92.9)	70.6(40.3, 100.0)	0.0010

a 95% CI was estimated with normal approximation.

b Fisher’s exact test.

**Table 3.** Comparison of pain NRS and Janda abdominal muscle strength scores between the two groups

	Baseline				8th week of treatment			
	Control (95%CI)	Treatment (95%CI)	Comparison (95%CI)	*p*	Control (95%CI)	Treatment (95%CI)	Comparison (95%CI)	*p*
Pain								
Lumbago	1.6(1.2, 2.1)	1.5(1.1, 2.0)	−0.1(−0.7, 0.5)	0.7232	1.3(1.1, 1.5)	0.1(−0.1, 0.3)	−1.2(−1.5, −0.9)	<.0001
Anterior abdomen	1.0(0.6, 1.4)	0.5(0.1, 0.8)	−0.5(−1.1, 0.0)	0.0649	0.6(0.4, 0.8)	0.3(0.1, 0.5)	−0.3(−0.6, −0.1)	0.0104
Janda Assessment	3.0(2.7, 3.3)	2.7(2.4, 3.0)	−0.3(−0.7, 0.1)	0.1845	3.4(3.2, 3.6)	4.4(4.2, 4.5)	1.0(0.8, 1.2)	<.0001

**Figure 5.**
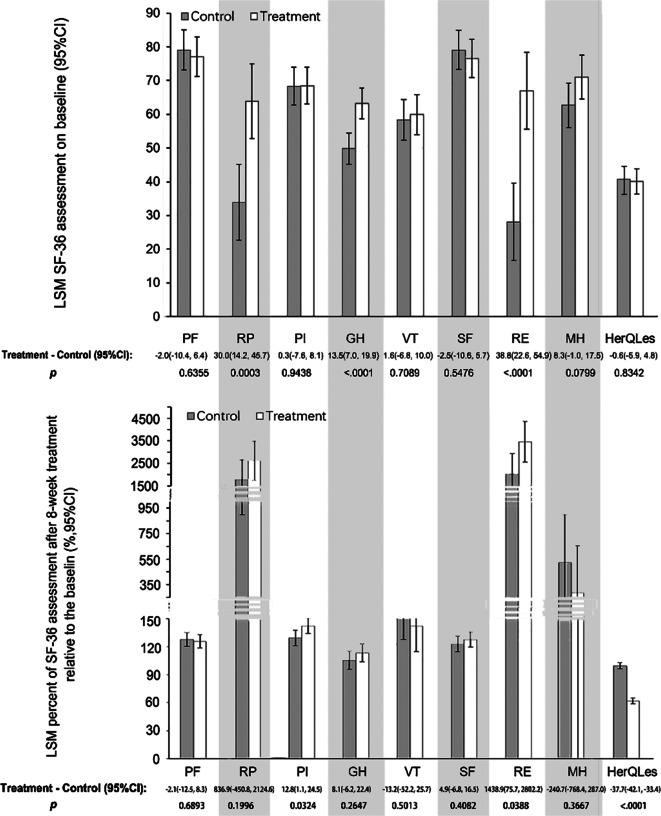
The LSM SF-36 assessment and HerQLes rank at baseline and the 8^th^ week of treatment. Above: the baseline score; Bottom: the percentage relative to the baseline. Abbreviations: LSM, least square mean; CI, confidence interval; PF, physical function; RP, role-physical; PI, pain; GH, general health; VT, vitality; SF, social functioning; RE, role-emotional; MH, mental health; HerQLes, Hernia-related Quality of Life Survey.

Regarding the SF-36 assessment, at the 8th week of treatment, the treatment group showed significantly higher increases in pain (PI) than the control group (142.2 [95% CI: 134.0–150.4]% versus 129.4 [121.1–137.7]%; the difference was 12.8 (1.1–24.5)% (*p* = 0.0324). Additionally, the treatment group showed significantly higher increases in the role-emotional (RE) scale (3,463.7 [2,560.3–4,367.0]% versus 2,024.7 [1,109.0–2,940.4]% than the control group; the difference was 1,438.9 (75.7–2,802.2)% (*p* = 0.0388). However, both groups demonstrated an increase in all SF-36 items in terms of the LSM percentage relative to the baseline at the 8^th^ week of treatment. Regarding HerQLes, the treatment group exhibited greater decreases in items from the baseline than those in the control group (61.9 [58.8–64.9]% versus 99.6 [96.5–102.7]%; the difference was −37.7 [−42.1 to −33.4]%, *p* < 0.0001) (Figure 5). The absolute changes for SF-36 subscales and HerQLes were shown in Supplementary Materials (Supplementary Table S1).

## 4. Discussion

DRA patients are characterized by abnormal abdominal wall muscle strength and anatomical structure changes. An earlier study has found that the application of NMES could augment the therapeutic effects of women with DRA (Kamel and Yousif, 2017). However, the study was mainly focused on patients with mild DRAM; the efficacy of NMES in moderate and severe DRAM patients needed to be further validated. One preliminary study applied shear wave elastography (SWE) to the evaluation of abdominal wall muscles in patients with W2, W3, as well as healthy controls, and reported that SWE propagated significantly lower in the RA and EO muscles of patients with DRA, whereas it propagated significantly higher in the TrA (He et al., 2021; Yuan et al., 2022; Zhou et al.,^2022^). Therefore, we stimulated the targeted sites, including bilateral EO, the upper and lower RA, for improving the primary weakness and laxity of shallow abdominal muscle groups with a trunk wearable NMES device. Furthermore, our research measured IRD with ultrasound, calculated the response rate of two groups, and analyzed the improvement of the trunk muscle strength, low-back pain as well as QoL to comprehensively compare the therapeutic efficacy between moderate and severe DRA patients, who received exercise therapy with or without the trunk-wearable NMES device treatment.

### 4.1. IRD (M3)

The clinical evaluation of DRA severity is mainly based on the IRD (van de Water and Benjamin, 2016; Reinpold et al., 2019). The M3 sector refers to the umbilical sector, which is the RA region 3 cm above and below the umbilical ring. Changes in the pregnancy process and anatomical structure of RA indicate that the IRD of the M3 sector is usually the most serious (Gilleard and Brown, 1996). Our results demonstrated that the IRD of the M3 sector was reduced in both the treatment and control groups; however, the reduction in the treatment group was significantly greater than that in the control group.

The IRD was negatively correlated with trunk flexor strength and trunk flexor endurance, and postpartum women who had greater IRD showed more decrease in trunk muscle strength (Liaw et al., 2011). The reduction in the IRD in the M3 sector in patients with DRA following exercise and NMES treatment may be closely related to the recovery of RA strength, the improvement of comprehensive TrA tension, and the relief of low-back pain (Benjamin et al., 2019). Our study was mainly based on physical therapy, which previously confirmed that its effect is of some clinical significance but has not reached the criterion of cure; therefore, we used the proportion of patients having at least one-level reduction in the IRD in the M3 sector for the primary efficacy assessment (as W3 to W2 and W2 to W1). The response proportion of patients with W2 and W3 in the control group was similar, whereas the response proportion of both patient classifications in the treatment group was much better than those in the control group. Additionally, patients with W3 in the treatment group had a significantly higher response proportion than those with W2, indicating that patients with a huge separation may have a better response proportion to the NMES. Owing to the flaccidity and dysfunction of the RA in patients with DRA, the nearby muscles might have contracted abnormally to compensate for the RA during training; thus, the intensity and effectiveness of the RA could not be guaranteed. The NMES device could accurately exert stimulation on the target muscles according to the treatment scheme, thereby leading to better improvement in the RA muscle strength and greater reduction in the IRD. Moreover, in the treatment group, patients with severe DRA had better efficacy than those with moderate DRA. This result further confirmed the significance of accurate stimulation of NMES and its importance for the recovery of muscle function, such as normal contraction, as patients with severe DRA presented with more serious dysfunction.

### 4.2. Trunk muscle strength

The function of trunk muscles, including strength, bearing, and immobilization of the abdominopelvic organs, would be frequently affected in women with DRA (Gunnarsson et al., 2015; Olsson et al., 2019). Liaw reported that women with DRA demonstrated significantly lower trunk muscle rotation torque at 6 months postpartum, and Hills showed that women with DRA had a lower capacity of trunk muscles at 1 year postpartum (Liaw et al., 2011; Hills et al., 2018). He et al. (2021) reported that the rectus RA and EO muscles of patients with DRA exhibit manifestations of disuse and atrophy. Furthermore, they found that the greater the IRD, the weaker the strength of the trunk muscles (Liaw et al., 2011). Our study showed that the trunk muscle strength of both groups before treatment decreased (completed the movement of neck flexion when the waist was on the table and lifted the inferior angle of the scapula, corresponding to levels two and three of the Janda assessment, respectively). The participants in the control group underwent exercise therapy, whereas those in the treatment group additionally had a trunk-wearable NMES device application. Compared with the previous study (Kamel and Yousif, 2017), our study showed that the trunk muscle strength of the control group had some improvements, whereas the therapeutic NMES schemes of the treatment group could significantly enhance the trunk muscle strength compared with the control group. NMES is a type of physical therapy that applies electric current to muscles and nerves to produce muscle contractions and simulate the exercise process, thereby promoting the recovery of muscle injury and preventing atrophy (Thabet and Alshehri, 2019). Moreover, trunk-wearable NMES device application could more accurately and appropriately stimulate the corresponding muscle groups, which compensates for the failure of using relevant muscles properly during exercise, thereby showing better efficacy than the control group (Kamel and Yousif, 2017). Moreover, our patients were instructed to use the wearable NMES device stimulation daily, which could provide convenience and improve their compliance, thereby ensuring the achievement of the required treatment intensity (Swanson et al., 2021); this may be one of the reasons why the trunk muscle strength of the treatment group was improved greater (Maffiuletti, 2010).

### 4.3. Low-back pain

DRA significantly reduces abdominal muscle strength, which affects the mechanical balance of low-back muscles, thereby resulting in a tilted pelvis, increased lumbar spine physiological curvature, and increased low-back pain incidence (Michalska et al., 2018). In our study, most patients in both groups had a certain degree of low-back pain (the rate was 71.1% [59/83]); after the 8-week treatment, the patients in the treatment group had much better pain relief than those in the control group. Ferreira *et al.* found that TrA plays a significant role in the occurrence of low-back pain (Ferreira et al., 2010). TrA muscle contraction was strengthened to maintain body balance and gradually formed compensatory muscle tension (He et al., 2021), which may have caused low-back pain and aggravated it based on midline instability in patients with DRA. Therefore, the significant improvement in low-back pain in the treatment group may be because the NMES scheme, which could accurately cause the relaxation and contraction of trunk muscle, rather than the compensatory pattern of the TrA. Additionally, NMES seems to cause temporary changes in cytokines, including interleukin (IL)-120 (Truong et al., 2017) and IL-10 (Brüggemann et al., 2017), which may explain the improvement in low-back pain in patients with DRA.

Furthermore, in our study, the NRS score of the control group decreased from 1.6 to 1.3 points, indicating that TrA-activating breathing exercises may help relieve low-back pain in patients with DRA partly for that activation, and the exercise of the TrA moved the bellies of the RA muscle together, improved the integrity of the linea alba, and increased fascial tension, thereby allowing efficient load transference and torque production (Lee et al., 2008). Moreover, the NRS score of the treatment group significantly improved owing to the NMES scheme that was targeted to the RA and EO muscles could enhance the abdominal wall muscle strength and subsequently relieve the load of the TrA (Hodges, 1999; Fukano et al., 2021; He et al., 2021), which may also play a critical role in alleviating low-back pain.

### 4.4. QoL

DRA affects women in multiple ways. Eriksson Crommert et al. (2020) conducted a study that reflected women’s experiences living with increased IRD following childbirth and reported that their body image, function, and strength deteriorated to different degrees after childbirth compared with those before childbirth. Emanuelsson et al. assessed SF-36 scores for eight domains encompassing physical wellbeing, emotional wellbeing, and other physical and emotional dimensions, and observed that these were lower in individuals with DRA than those of age-matched controls (Emanuelsson et al., 2016). Our study showed that the treatment group had greater improvement in the QoL assessed using the SF-36 questionnaire than the control group in terms of PI and RE, and the improvement could also be observed in HerQLes. This may be achieved by low-back pain reduction and trunk muscle strength enhancement. The QoL assessment further suggests that adding trunk-wearable NMES to the DRA rehabilitation program will be valuable for the recovery of patients with DRA.

### 4.5. Age and fetal weight

In terms of the baseline subject characteristics, we found that there is a significant difference in age between the control and intervention groups (*p* = 0.0459) as well as fetal weight (*p* = 0.0393). According to previous studies, due to individual differences and factors such as diet and exercise, the minor age differences among postpartum women DRA may not lead to noticeable variations in relevant muscle anatomical structures and functions, but the treatment efficacy may differ between young and elderly postpartum women for the aging-related decline in skin elasticity and muscle fibers (Radhakrishnan and Ramamurthy, 2022; Sartori et al., 2024). Apart from that, the sample size and concentrated age distribution may be the potential reasons for this condition in our research. These findings suggest that we should expand the sample size to explore in depth the correlation between changes in neonatal weight, fetal position, and occurrence and classification of DRA, which may provide evidence for careful weight management during pregnancy, predicting the occurrence of DRA in different fetal positions and thus making early prevention of DRA and prenatal preparations, and further investigate age-related changes in postpartum women with DRA, as well as comparative analyses of rehabilitation outcomes in the future study.

### 4.6. Clinical implications

This work firstly reveals that the effect of a trunk-wearable NMES device accompanied by core exercise programs in patients with moderate and severe diastasis, and by comparing the effect with patients who received exercise only, we provide clinical evidence for a new perspective non-invasive strategy for moderate and severe DRA patients.

### 4.7. Limitations

The treatment cycle was 8 weeks long. Porcari JP et al. have found that the use of the NMES belt on adults for 8 weeks could significantly improve the muscular strength and endurance of the abdominal region, and improved self-perceived abdominal firmness and tone (Porcari et al., 2005). Kamel and Yousif (2017) have demonstrated that 8 weeks EMS treatment could potentially be a benefit for postpartum women with DRA. Based on the current results of our study, an 8 weeks Trunk-Wearable NMES device treatment has shown positive effects in improving IRD of patients with moderate and severe DRA, greater response proportion, improving low back pain and quality of life. In subsequent studies, we will further explore the optimal treatment duration for the trunk-wearable NMES device in patients with moderate and severe DRA. Apart from that, A 6-month follow-up study with the aim of investigating the natural recovery of IRD and abdominal muscle function has shown that the IRD and abdominal muscle function of postpartum women improved but had not returned to normal values at 6 months after childbirth (Liaw et al., 2011). Coldron et al. (2008) also reported that most of the natural recovery of IRD occurred by 8 weeks, but no further improvement was noted at the end of the first year, suggesting that partial recovery of DRA happens after childbirth but is incomplete even after 1 year. Besides, Abdelaziz et al. (2021) have found that EMS can potentially be of benefit in treating DRA compared to natural healing. The effect is more evident in the first month postpartum and decreases with time. Therefore, these studies indicated that we should pay attention to the spontaneous recovery and include a natural recovery group for comparative analysis. In subsequent studies, we will extend the follow-up periods to assess the long-term effects of the wearable NMES device on diastasis recti and other abdominal muscles in patients with moderate and severe DRA through ultrasound and electromyography examinations, as well as compare the effects with natural healing.

## 5. Conclusion

The application of trunk-wearable NMES devices in patients with moderate and severe DRA can reduce the IRD, and patients responded better than those who received exercise therapy only. Moreover, the application of an NMES device accompanied by exercise therapy can significantly improve trunk muscle strength, reduce low-back pain, and enhance the QoL of patients with DRA.

## Supporting information

Zheng et al. supplementary material Zheng et al. supplementary material

## Data Availability

The data that support the findings of this study are available from the corresponding author upon reasonable request.

## Supplementary material

The supplementary material for this article can be found at http://doi.org/10.1017/wtc.2025.10035.
