# Supplementary material for: Effectiveness of a trunk-wearable neuromuscular electrical stimulation device in postpartum women with diastasis rectus abdominis: A prospective randomized controlled trial
Source: Wearable Technol. 2025 Dec 11;6:e55. doi: 10.1017/wtc.2025.10035 (PMC12724349; doi:10.1017/wtc.2025.10035)
Supplement: Zheng et al. supplementary material [file S2631717625100352sup001.zip › Table_S1.docx]

|  | Control | Treatment | *p* Value^a^ | *p* Value^b^ |
| --- | --- | --- | --- | --- |
| N | 41 | 42 |  |  |
| PF, Mean±SD | 13.1± 23.74 | 15.0± 18.97 | 0.680 | 0.936 |
| RP, Mean±SD | -37.2± 52.18 | 30.4± 41.15 | ＜0.001 | ＜0.001 |
| PI, Mean±SD | 12.9± 20.46 | 18. 8± 18.98 | 0.178 | 0.008 |
| GH, Mean±SD | 4.0± 17.30 | -4.9± 16.18 | 0.018 | 0.033 |
| VT, Mean±SD | 9.2± 25.12 | 11.2± 26.67 | 0.720 | 0.211 |
| SF, Mean±SD | 10.5± 19.07 | 14.6± 20.77 | 0.344 | 0.398 |
| RE, Mean±SD | -48.8± 45.40 | 28.6± 38.67 | ＜0.001 | ＜0.001 |
| MH, Mean±SD | 13.9± 27.44 | 5.2± 22.80 | 0.122 | 0.878 |
| HerQLes, Mean±SD | -1.1± 31.23 | 8.6± 27.26 | 0.135 | 0.009 |
| M1 (mm), Mean±SD | -2.9± 4.10 | -2.0± 4.76 | 0.366 | 0.480 |
| M2 (mm), Mean±SD | -6.4± 6.15 | -5.4± 7.67 | 0.491 | 0.845 |
| M3 (mm), Mean±SD | -7.8± 5.37 | -12.9± 9.98 | 0.005 | 0.003 |
| M4 (mm), Mean±SD | -3.0± 6.66 | -4.9± 7.28 | 0.219 | 0.193 |
| M5 (mm), Mean±SD | -0.3± 1.61 | -1.0± 3.05 | 0.163 | 0.018 |
| Janda Assessment scores, Mean±SD | 0.5± 0.60 | 1.6± 0.83 | ＜0.001 | ＜0.001 |

Table S1. Comparison of SF-36, HerQLes, IRD and Janda assessment changes between the two groups

^a^: Student’s *t*-test; ^b^: Univariate Analysis of Variance, adjusted for baseline differences between groups (age, fetal weight). PF, physical function; RP, role-physical; PI, pain; GH, general health; VT, vitality; SF, social functioning; RE, role-emotional; MH, mental health; HerQLes: Hernia-related Quality of Life Survey; M1 = subxiphoidal sector; M2 = epigastric sector; M3 = umbilical sector; M4 = infraumbilical sector; M5 = suprapubic sector.
