# Supplementary figures and images for: Effectiveness of a trunk-wearable neuromuscular electrical stimulation device in postpartum women with diastasis rectus abdominis: A prospective randomized controlled trial
Source: Wearable Technol. 2025 Dec 11;6:e55. doi: 10.1017/wtc.2025.10035 (PMC12724349; doi:10.1017/wtc.2025.10035)

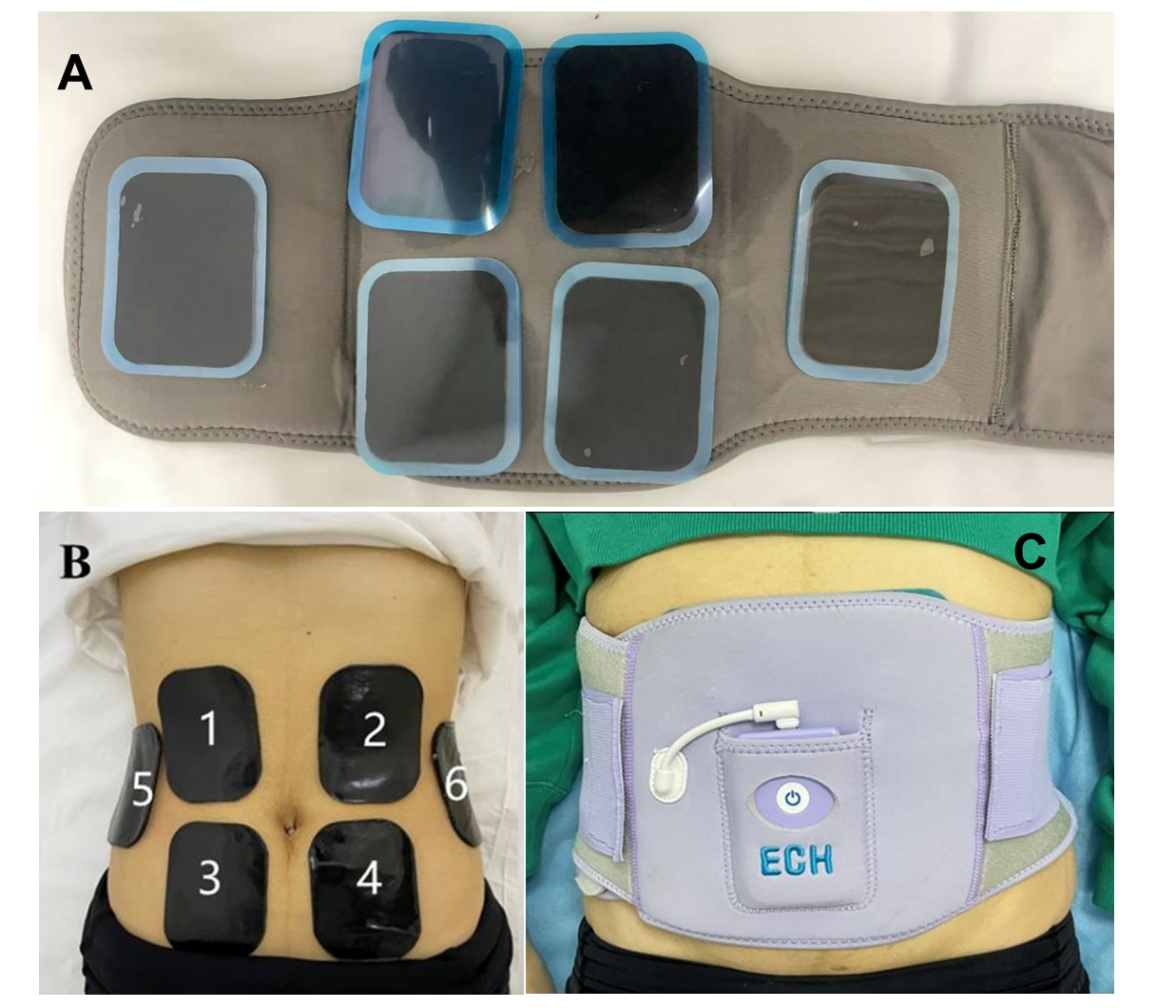

Supplement: Zheng et al. supplementary material [file S2631717625100352sup001.zip › Fig._S1.tif]
